# Supplementary material for: Active site specificity profiling datasets of matrix metalloproteinases (MMPs) 1, 2, 3, 7, 8, 9, 12, 13 and 14
Source: Data Brief. 2016 Feb 22;7:299–310. doi: 10.1016/j.dib.2016.02.036 (PMC4777984; doi:10.1016/j.dib.2016.02.036)
Supplement: Supplementary file 10 — Supplementary material [file mmc10.zip › WebPICS_hMMP14_T_1%/subsite_coop.html]

 

PICS results


- # About
- # Manual
- # Analysis
- # Results

- Redraw
- Seqlogo
- Dependency
- Coop

**Potential subsite cooperativity analysis for hMMP14\_T\_1%**   
Minimum difference for subsite dependency was set to +- 10 percentage-points  
Subsite dependency was checked for all positional occurences above 1 x natural abundance  
**Subsite dependency check is restricted to P3 - P3'**

|  |  |  |  |
| --- | --- | --- | --- |
| Fixed residue | Affected residue(s) | Change (percentage-points) | Vice-Versa change |
| P3\_A | P2\_N | 15.8 | 23.2 |
| P3\_H | P1\_D | 31.0 | 12.4 |
| P3\_H | P2prime\_M | 20.1 | 10.7 |
| P3\_I | P2\_Q | 17.0 | 17.0 |
| P3\_I | P2\_S | 16.3 | 14.9 |
| P3\_L | P2prime\_I | 15.3 | 13.1 |
| P3\_S | P1prime\_Q | 14.4 | 21.8 |
| P3\_S | P2\_E | 16.3 | 14.9 |
| P3\_S | P3prime\_V | 15.3 | 12.4 |
| P3\_V | P1prime\_M | 12.7 | 16.3 |
| P2\_A | P2prime\_L | -11.5 | -15.4 |
| P2\_C | P1prime\_Q | 20.4 | 17.5 |
| P2\_E | P1\_D | 10.9 | 12.5 |
| P2\_M | P1\_F | 15.8 | 12.1 |
| P2\_N | P1prime\_V | 22.2 | 13.8 |
| P2\_Q | P2prime\_I | 26.7 | 16.1 |
| P2\_Q | P3prime\_D | 18.3 | 22.6 |
| P1\_C | P1prime\_Q | 24.0 | 12.0 |
| P1\_C | P3prime\_N | 23.4 | 10.2 |
| P1\_G | P1prime\_W | 12.5 | 57.1 |
| P1\_M | P2prime\_W | 10.9 | 17.4 |
| P1\_N | P3prime\_L | 19.0 | 21.2 |
| P1\_P | P3prime\_S | 12.9 | 14.0 |
| P1prime\_Q | P2prime\_Q | 27.6 | 15.4 |
| P1prime\_W | P2prime\_H | 12.5 | 11.5 |
| P2prime\_C | P3prime\_Q | 45.8 | 21.1 |
| P2prime\_M | P3prime\_V | 18.2 | 10.5 |

  
**PICS analysis of protease: hMMP14\_T\_1%**  
307 cleavage sites analyzed  
PICS library made with (T)rypsin, (G)luC or (C)hymotrypsin:   
Cutoff for graphic display: 2 x natural abundance  

|  |  |
| --- | --- |
| Positional occurences  (table for total and relative (in %) values) | Occurences relative to natural abundance (table) |
|  |  |
